# Domestic Servants

**Published:** 1871-04

**Authors:** 


### DOMESTIC SERVANTS

Are human beings. This announcement may surprise some who read it, but it is true for all that. Half the housewives in the land, at the very least one half of them, have forgotten this important truth, if, indeed, they ever knew it.

Domestic servants are full of human nature; in fact, they are a great deal too full of it; and the only remedy is to take out somé, and replace it with grace; that is, let mistresses, instead of heaping upon them all the epithets of the "catalogue," as Dame Partington would say, lift them to a higher plane of life, by considerate courtesies and humane forbearance. They are ignorant, and allowance should be made for it. They have feelings, and those feelings should be respected. They need encouragement; they need a good example. Uniform civility and politeness to servants is a power over them. Servants, with all their want of learning and with all their disadvantages of birth and rearing, are quick and accurate observers of character; and they fix in their own mind the moral status of the mistress, even a little quicker than the mistress forms a true estimate of their availability as aids in housekeeping; and whatever they see which lowers their estimation of the mistress, diminishes their respect for her, and makes them less ambitious to please her and to secure her good-will.

If the mistress is passionate and impatient, if she is changeable, if she has no mind of her own, if she is double-faced, has one bearing towards visitors when present and another when they are gone, if petulant at the table, if unreasonable in her exactions, if inconsiderate in the amount of services required, if, in a measure, regardless of the personal comfort and welfare of those in the kitchen, every deficiency is measured, and the standard of respect lowered accordingly.

Scolding, loud talking, depreciating epithets, never made any servant better, always makes them more inefficient and unreliable; it is much better to appeal to their intelligence, their self-respect, seldom, if ever, to their religion; that is too sacred a thing to be brought into the daily affairs of common life.

Familiarity with servants is always a mistake; keep them at a respectful distance; let them be made to feel your superiority, not by mere assertion, but by your high bearing. Encouragement, courtesy, patience, consideration, and sympathy, these are the qualities which will seldom fail to make bad servants good, and good servants better, especially when the maid sees that the mistress knows how things ought to be done. Domestic rule should be one of love rather than of fear.

